# Relationship of Brain Glutamate Response to D-Cycloserine and Lurasidone to Antidepressant Response in Bipolar Depression: A Pilot Study

**DOI:** 10.3389/fpsyt.2021.653026

**Published:** 2021-06-02

**Authors:** Zhengchao Dong, Michael F. Grunebaum, Martin J. Lan, Vashti Wagner, Tse-Hwei Choo, Matthew S. Milak, Tarek Sobeih, J. John Mann, Joshua T. Kantrowitz

**Affiliations:** ^1^Department of Psychiatry, College of Physicians and Surgeons, Columbia University, New York, NY, United States; ^2^Molecular Imaging and Neuropathology, New York State Psychiatric Institute, New York, NY, United States; ^3^Mental Health Data Science, New York State Psychiatric Institute, New York, NY, United States; ^4^Information Sciences, Nathan Kline Institute, Orangeburg, NY, United States; ^5^Department of Radiology, College of Physicians and Surgeons, Columbia University, New York, NY, United States; ^6^Psychotic Disorders, New York State Psychiatric Institute, New York, NY, United States; ^7^Information Sciences, Nathan Kline Institute, Orangeburg, NY, United States

**Keywords:** N-methyl-D-aspartate, glutamate, MRS—^1^H nuclear magnetic resonance spectra, biomarker, bipolar depression, D-Cycloserine, lurasidone

## Abstract

N-methyl-D-aspartate glutamate-receptor (NMDAR) antagonists such as ketamine have demonstrated efficacy in both major depressive disorder (MDD) and bipolar disorder depression (BP-D). We have previously reported that reduction in Glx (glutamate + glutamine) in the ventromedial prefrontal cortex/anterior cingulate cortex (vmPFC/ACC), measured by proton magnetic resonance spectroscopy (^1^H MRS) at 3T during a ketamine infusion, mediates the relationship of ketamine dose and blood level to improvement in depression. In the present study, we assessed the impact of D-cycloserine (DCS), an oral NMDAR antagonist combined with lurasidone in BP-D on both glutamate and Glx. Subjects with DSM-V BP-D-I/II and a Montgomery-Asberg Depression Rating Scale (MADRS) score>17, underwent up to three ^1^H MRS scans. During Scan 1, subjects were randomized to receive double-blind lurasidone 66 mg or placebo. During Scan 2, all subjects received single-blind DCS 950 mg + lurasidone 66 mg, followed by 4 weeks of open label phase of DCS+lurasidone and an optional Scan 3. Five subjects received lurasidone alone and three subjects received placebo for Scan 1. Six subjects received DCS+lurasidone during Scan 2. There was no significant baseline or between treatment-group differences in acute depression improvement or glutamate response. In Scan 2, after a dose of DCS+lurasidone, peak change in glutamate correlated negatively with improvement from baseline MADRS (r = −0.83, *p* = 0.04). There were no unexpected adverse events. These preliminary pilot results require replication but provide further support for a link between antidepressant effect and a decrease in glutamate by the NMDAR antagonist class of antidepressants.

## Introduction

Bipolar disorder affects 2% of the population in the United States (1). Despite overall effectiveness of FDA approved compounds, many individuals with bipolar depression (BP-D) experience persistent depression despite antidepressant medication treatment, either alone or combined with mood stabilizers. For example, across several recent registration studies, ~40-50% of subjects were non-responders based upon Montgomery-Asberg Depression Rating Scale (MADRS) (2) scores ≥50% of baseline (3–5).

Recently, the N-methyl-D-asparate glutamate-receptor (NMDAR) antagonist, ketamine, has emerged as a potential treatment option for both major depressive disorder (MDD) (6, 7) and (BP-D) (8). Although the antidepressant mechanism of action of ketamine remains unclear, convergent evidence suggests that dysfunction of glutamatergic systems plays a role in the pathophysiology of BP-D (9, 10).

However, intravenous ketamine use is limited by loss of benefit after about 5–7 days and transient psychotomimetic side effects during administration. Intranasal ketamine is easier to administer but may have more side effects (11, 12). d-cycloserine (DCS), an FDA-approved anti-tuberculosis drug, is an NMDAR antagonist at higher doses. It is primarily an antagonist at >500 mg (13–15), via the glycine co-receptor of the NMDAR and may have a more favorable safety profile than ketamine. Potential antidepressant effects of DCS were first reported in 1959 (16) but not formally studied until recently. Efficacy of DCS in a dose of >500 mg in MDD, including an anti-suicidal effect, is supported by two double-blind studies (15, 17). Recently, we reported an open label study of treatment resistant BP-D—a single infusion of ketamine followed by 8 weeks of a combination of DCS + FDA approved medications for BP-D (including lurasidone). This combination was employed seeking a treatment where an atypical antipsychotic prevented any potential psychomimetic effect of DCS and perhaps had an additional antidepressant action. Indeed, a sustained benefit for the duration of treatment was seen (46% symptom reduction, *p* = 0.019 vs. baseline) without significant safety concerns (18). Of note, there was a decline in benefit over the first 2 weeks post ketamine, that reversed with the ongoing combination of DCS and lurasidone or other FDA-approved treatments for BP-D.

In previous studies, we used proton magnetic resonance spectroscopy (^1^H MRS) to quantify ketamine effects on Glx (a combination of glutamate (Glu) and glutamine resonance signals: Glu+glutamine) in the ventromedial prefrontal cortex, along with the adjacent anterior cingulate cortex (vmPFC/ACC) in both healthy (19) and depressed (20, 21) individuals. Our focus on the vmPFC and the ACC stems from extensive research implicating these regions in the pathogenesis of mood disorders (22–24) and microdialysis rodent (25, 26) studies suggesting that the glutamatergic surge in response to NMDAR antagonists is maximal in the vmPFC.

In our recently published, placebo-controlled, dose-finding, randomized clinical trial of ketamine (21), we found that improvement in MDD had a positive linear relationship with ketamine dose and blood level, and a negative correlation with Glx response. Reduction of Glx mediated the relationship of ketamine dose and level with antidepressant response. In the present report, we sought to determine whether the same relationship is found within BP-D for DCS combined with lurasidone. This combination of medications seeks to preserve the antidepressant effect of DCS and block its potential psychotomimetic effect with lurasidone. Lurasidone may also augment the antidepressant effect of DCS since it an FDA approved medication for BP-D (3, 4).

To determine the independent biological effect of lurasidone, prior to the DCS/lurasidone scan, all subjects underwent an ^1^H MRS scan while receiving double-blind lurasidone 66 mg or placebo. We have previously shown that this ^1^H MRS method is sensitive to DCS-induced changes in Glx in healthy controls (27). Due to upgrades in both scanner quality and ^1^H MRS methodology (28–30), we now report the more specific ^1^H MRS outcome of Glu, in place of Glx. We hypothesized that we would find a similar relationship between Glu and DCS+lurasidone mediated antidepressant response, thus adding to our understanding of NMDAR antagonist mechanism of action in depression.

## Patients and Methods

The study was conducted under a biomarker letter of support (IND 129194) from the US Food and Drug Administration and posted on www.clinicaltrials.gov (NCT03402152).

Enrollment criteria included DSM-V current BP-D-I/II, confirmed by a SCID (31). Subjects had at least moderate depression symptoms, as defined by a MADRS score>17, with no current or chronic psychosis or substance use disorder. To minimize further acute clinical deterioration, subjects were permitted to remain on all current pre-study psychotropics, with the exception that prior antipsychotics and fluoxetine were discontinued at least 24 h before the first MRI to mitigate the effect of such medications on the Glu response to acute administration of DCS or lurasidone.

After screening, each eligible subject underwent up to three ^1^H MRS scans, on three different days (referred to as Scan 1, Scan 2, and Scan 3, respectively). During Scan 1, all subjects were randomized to receive double-blind lurasidone 66 mg or placebo, and during Scan 2 all subjects received single-blind one dose of NRX-101 (DCS 950 mg + lurasidone 66 mg). All subjects received a dose of pyridoxine (200 mg) along with the study medication to prevent DCS related reductions in Vitamin B6 (32). After Scan 2, subjects were started on a combination of open-label flexibly dosed DCS/lurasidone and daily pyridoxine 200 mg for 4 weeks, culminating in an optional final ^1^H MRS scan (Scan 3).

Scans 1 and 2 were at least 1 day apart, and subjects and data analysts, including ^1^H MRS data processing, were blind to treatment order (e.g., unaware that DCS+lurasidone was always administered immediately prior to Scan 2). The mean time between Scans 1 and 2 was 3.3 days (range 1–7 days). After structural MRI and baseline ^1^H MRS scans (~30 min), subjects were briefly removed from the scanner for study drug administration, followed by serial ^1^H MRS frame acquisitions for up to 70 min following drug administration. Subjects were assessed using a side effects checklist, the C-SSRS and MADRS at baseline, ~30 min before and after the imaging on the ^1^H MRS days and weekly during the 4-week follow-up.

The study was terminated by the sponsor after eight randomized subjects due to a corporate decision to pursue a different approach. Thus, we only report pilot results due to the limited sample size.

### ^1^H MRS Methodology

**Figure 1:**
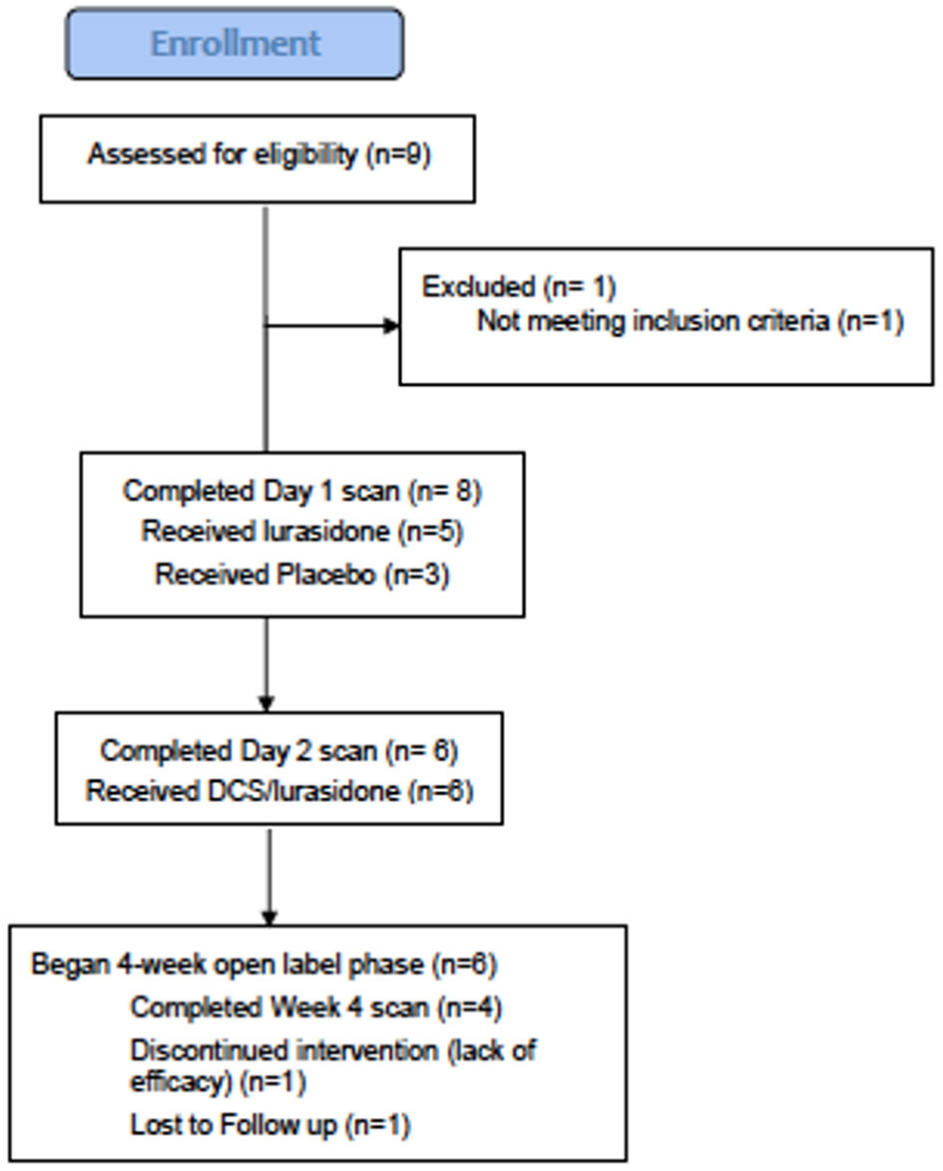
CONSORT chart.

Six subjects were scanned on a Siemen Prisma 3.0T MR scanner equipped with a 32-channel surface coil array and two subjects were scanned on a General Electric SIGNA Premier 3T MR scanner equipped with a 21-channel surface coil array. MR data were acquired with the same protocols on both scanners. The protocols for voxel placement and ^1^H MRS data acquisition for both sessions of before and after medication were the same. First, three-plane scout images were acquired, followed by a high-resolution structural MRI scan in the sagittal planes; Then, high resolution structural MRI images in the oblique axial planes parallel to the AC-PC line were acquired. We placed the ^1^H MRS voxel (3.0 × 2.5 × 2.5 cm^3^) based on the sagittal and axial MR images in the vmPFC and ACC, with the center of the posterior side of the voxel close to the frontal tip of the cingulate gyrus (Figure 1). The ^1^H MRS data were acquired from the voxel using a commercial version of the PRESS sequence (33) implemented on both scanners with following parameters: TR/TE = 1,500/120 ms (28, 29), spectral width = 2,000 Hz, free induction decay (FID) datapoints = 1,024, number of excitations (NEX) for water unsuppressed ^1^H MRS scan = 16, and NEX for water suppressed ^1^H MRS = 240. Total scan time for each ^1^H MRS frame, including pre-scan, was ~8 min.

### ^1^H MRS Data Processing

We combined the multichannel ^1^H MRS data, using the unsuppressed water signal as a reference for correcting phase errors and for calculating weighting factors of *S/N*, where *S* is the amplitude of water signal and *N* is the standard deviation of noise of each channel (34). We then corrected frequency and phase shifts among the FIDs in each ^1^H MRS data file and combined them into a single FID for each baseline and dynamic ^1^H MRS scan. We removed the residual water signal using a singular value decomposition-based matrix-pencil method (35).

We quantified the ^1^H MRS data in the time domain using the software packages AMARES (36) imbedded in jMRUI (37). To improve accuracy of the quantification of the metabolites of interest via spectral fitting, we fit all peaks with major contributions, including metabolic peaks of N-acetylaspartate at 2.01 ppm, total creatine (Cr) at 3.02 ppm, total choline at 3.24 ppm and Glu around 2.26 ppm were included in the spectral fitting. In the present paper, we focus on the role of Glu/Cr and Glx/Cr, and did not analyze NAA and Ch. For accurate spectral fitting of Glu, we incorporated prior knowledge in the model of relative frequencies, phases, and amplitudes of the major peaks Glu obtained by fitting the simulated spectra of Glu using AMARES, similar to the approach in the reference (38), where the prior knowledge was obtained from phantom ^1^H MRS data of Glu. The simulations of Glu spectra for both Siemens and GE data were performed using the MARSS software package (30).

Due to higher quality measurements on our upgraded scanner, we modified the initial analysis plan posted on clinicaltrials.gov, and utilized Glu peak, as opposed to Glx AUC, as the primary metabolite outcome. Glx was analyzed as a secondary measure. Based on pharmacokinetics (39–41) of DCS, our prior finding of the ^1^H MRS peak at ~35 min DCS post-dose (27) and our prior ketamine study using peak level (21), Glu ^1^H MRS peak level was used, defined as a mean from 30 to 46 min post drug. We used Cr as a reference for the relative quantification of Glu and expressed the outcome measure from ^1^H MRS as Glu/Cr. The rationale for using Cr as a standard is as follows: (1). The Cr level is assumed to be stable over the course of drug administration; and (2). The tissue volumes for Glu and Cr are the same in the voxel and a partial volume effect of using water as a reference is avoided. We used the ratio of standard deviation to estimated amplitude given in the fitting by the jMRUI software, as a metric for quality control and set the threshold to be 20%. No data were excluded (42).

### Data Analysis

Prior to analysis, all variables were examined for distribution and outliers. Due to the small sample sizes, parametric tests were utilized only for repeated-measures modeling of change in MADRS, for which residuals were sufficiently normal.

Wilcoxon sign rank tests were used to test for significant Scan 1 percent change in MADRS and Glu within treatment group, and Scan 2 percent change in MADRS and Glu in the overall sample. Additionally, Wilcoxon sign rank tests were also used to assess both baseline and change in MADRS and Glu response from Scan 1 to Scan 2, within subject.

Mixed effects linear regression models were fit to model MADRS change from baseline over the four follow-up weeks. First, an intercept-only model was fit to assess mean change across the 4 weeks. Next, week was added to the model as a categorical predictor to estimate change from baseline at each week. Both models featured a random intercept for subject. Spearman's correlations were used to assess the association between MADRS and Glu responses, on Scan 1 and on Scan 2, separately. Due to the small sample sizes, descriptive statistics are provided in the text.

Significance level was set at α = 0.05, with results reported as mean ± standard deviation (SD) and median with interquartile ranges (IQR, 25th percentile, 75th percentile). All analyses were performed using SAS version 9.4 (Cary, NC: SAS Institute Inc.; 2014). The data that support the findings of this study are available on request from the corresponding author if accompanied by a reasonable plan for their use. The data are not publicly available due to privacy or ethical restrictions.

## Results

**Figure 2:**
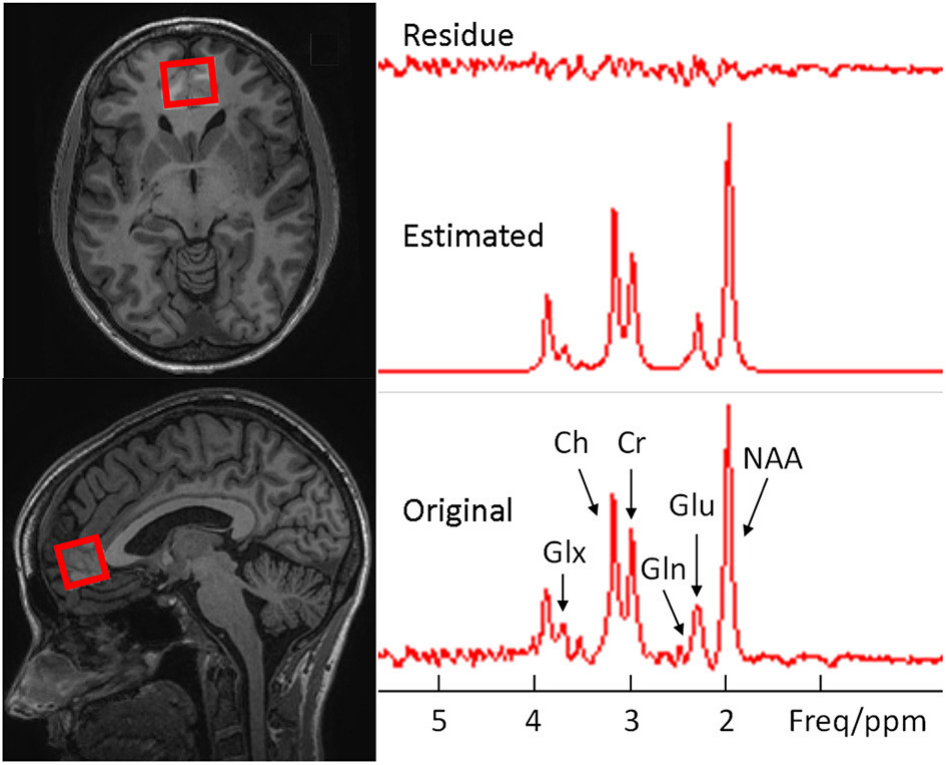
Examples of voxel placement (red outline) on the axial and sagittal localizer images showing the size and location in the medial ventral prefrontal cortex (Left) and the original, estimated, and their difference spectra from the voxel (Right).

Subjects: 9 subjects consented to participate (Figure 2), eight met study criteria and were randomized (Table 1). On entering the study, three randomized subjects were unmedicated, and the remaining five randomized subjects were on stable doses of mood stabilizers and antidepressants for at least 1 month, including one subject on oxcarbazepine 600 mg and escitalopram 20 mg, one subject on lithium 450 mg and fluoxetine 60 mg (discontinued prior to scan), one subject on lamotrigine 50 mg and diphenhydramine 50 mg, one subject on sertraline 200 mg, zolpidem 10 mg, gabapentin 1,000 mg and diazepam 10 mg and one subject on valproic acid 1,000 mg, paroxetine 20 mg and dextroamphetamine and amphetamine 20 mg. Six out of eight randomized subjects completed the first two scans, and both non-completers received lurasidone during Scan 1. Four subjects completed the four-week open-label phase, with ^1^H MRS available for three subjects.

**Table 1:** Baseline demographics, psychopathology and subject disposition.

Age (years)	32.4 ± 13
Sex	7 women
Diagnosis	Bipolar I (*n =* 5) Bipolar II (*n =* 3)
Medications	Mood stabilizer alone (*n =* 3), SSRI + Mood stabilizer (*n =* 2), Unmedicated (*n =* 3)
Illness duration (months)	19.7 ± 34.8
Hospitalizations (*n*)	1.5 ± 1.4
Manic/hypomanic episodes (*n*)	5 ± 6.1
MDD episodes (*n*)	5.9 ± 8.8
Baseline MADRS (screening)	31.5 ± 9.3
Baseline C-SSRS	2 ± 2
Received lurasidone (Scan 1)	*N =* 5
Received placebo (Scan 1)	*N =* 3
Received D-Cycloserine/lurasidone (Scan 2)	*N =* 6
Pre-scan MADRS (Scan 1)	25.3 ± 7.2
Post-scan MADRS (Scan 1)	11.3 ± 8.0
Pre-scan MADRS (Scan 2)	20.0 ± 11.2
Post-scan MADRS (Scan 2)	11.3 ± 10.0

### Clinical

Five subjects were randomized to lurasidone and three to placebo on Scan 1. Overall, subjects exhibited a comparable degree of acute improvement from the baseline MADRS after one dose of lurasidone alone (57.0% ± 31.7, *p* = 0.06, *n* = 5) or placebo (72.7% ± 32.6, *p* = 0.25, *n* = 3) at Scan 1; and sustained this improvement after a mean of 3.3 days of one dose of DCS+lurasidone at Scan 2 (67.2% ± 22.6, *p* = 0.03, *n* = 6). Only the DCS+lurasidone improvement at Scan 2 relative to baseline reached statistical significance. Among subjects that completed both scans, there was no significant difference between baseline MADRS on scan days, suggesting a lack of carryover effect from Scan 1 (Signed-Rank Test *p* = 0.31).

**Figure 3:**
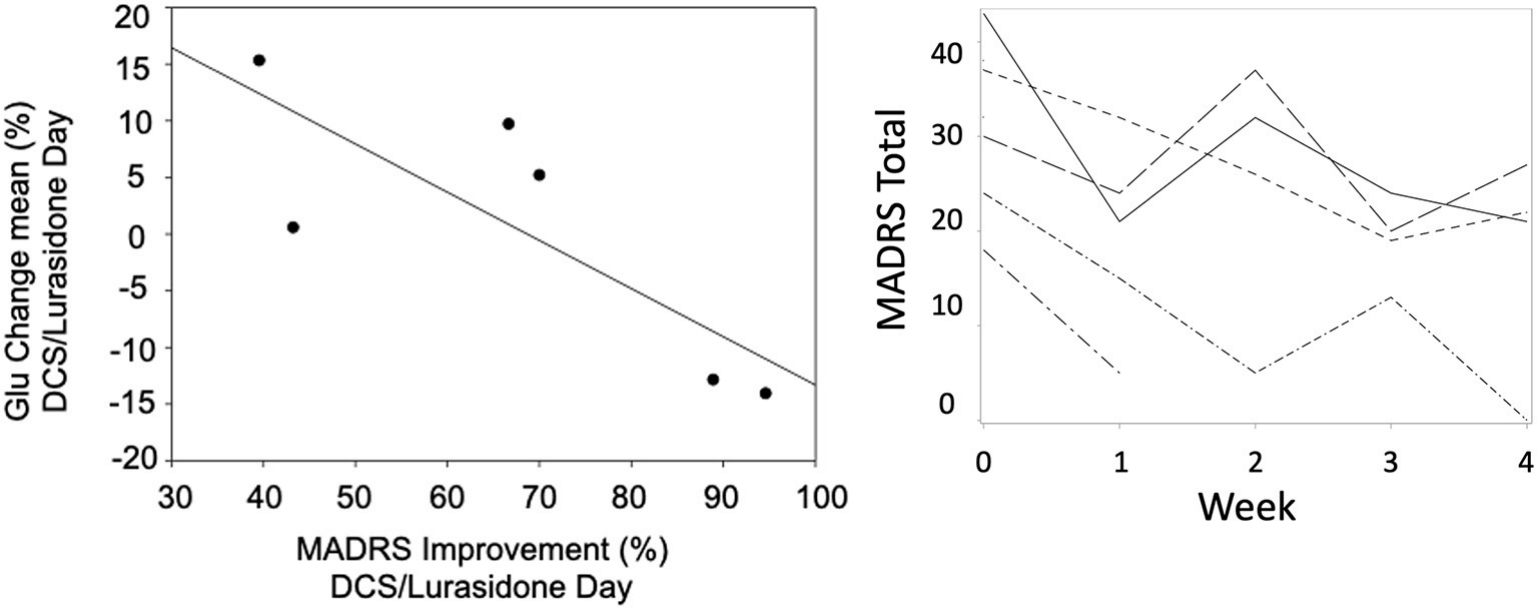
A scatter plot of mean change in Glu vs. improvement from baseline MADRS (r_s_ = −0.83, *p* = 0.04) on the DCS/Lurasidone days (Left). Spaghetti plot of MADRS over time by subject (Right).

Using mixed-effects linear modeling, a significant overall MADRS improvement over time was seen (t_4_ = −6.38, *p* = 0.0031) over the 4-week treatment, with the final MADRS total decreasing to 17.5 ± 12.0. Weekly contrasts demonstrated significant improvement from baseline at all rating points except at 2 weeks (*p* = 0.0038, Figure 3 Right). No subjects achieved euthymia, defined by MADRS <8.

No patients exhibited active suicidal ideation, intent or behavior during the study on the C-SSRS (all C-SSRS scores <3). There were no unexpected side effects. There was one serious adverse event involving a patient who was observed overnight in hospital for moderate dystonia thought to be related to lurasidone. This subject remained in the study with a reduction in dose.

^1^H MRS: As previously (27), the Glu peak was ~35 min post oral drug administration. Consistent with our previous work with ketamine in MDD (21), Glu increase was seen after placebo only. Both lurasidone alone and the DCS+lurasidone combination attenuated the Glu response. However, neither the within nor the between group changes in Glu levels were significant statistically. The changes observed were small: a decrease after lurasidone [Median (IQR) = −6.6% (−16.9%,−1.9%), *n* = 5, *p* = 0.31]; and increase after placebo [Median (IQR)= +12.9% (−9.0%,34.8%), *n* = 3, *p* = 1.0]; and a decrease after DCS+lurasidone treatment [Median (IQR)= −2.7% (−7.6%,−2.2%), *n* = 6, *p* = 0.31] on the ^1^H MRS scan days (Wilcoxon sign rank test). Within the same subjects, Glu response decreased after DCS+lurasidone and lurasidone alone (median: −3.9% vs. −7.4%, *n* = 4) and increased after placebo (median: 20.3% vs. −6.7%, *n* = 2) but none of these effects were statistically significant.

On the DCS+lurasidone treatment day (Scan 2), change of Glu from baseline on that day correlated negatively with improvement from baseline MADRS (r_s_ = −0.83, *p* = 0.04, Figure 3, Left), using Spearman's correlation coefficient. By contrast, on the placebo/lurasidone day (Scan 1), Glu change did not correlate with MADRS change (r_s_ = 0.29, *p* = 0.53). Controlling for Scan 1 treatment-type did not change results for Scan 1 or 2 correlations. There were no significant differences in baseline Glu levels on Scan 1 and Scan 2 [Day 1 Med (IQR) = 0.31 (0.20–0.35); Day 2 Med (IQR) = 0.29 (0.22–0.37); Signed-Rank Test *p* = 0.69], supporting the assumption that this relationship was not due to carryover effects from Scan 1. Only 3 subjects completed Scan 3 (Week 4) ^1^H MRS, without significant results.

Glx did not show any statistically significant results in Day 1 or Day 2 analysis. Change in Glx on Day 2 did not correlate with either change in Glu on Day 2 (r_s_ = 0.54, *p* = 0.27), nor with MADRS change (r_s_ = −0.37, *p* = 0.47).

## Discussion

Due to the small sample size, particularly for the placebo and lurasidone alone groups, the present findings are presented as a preliminary pilot study. Nevertheless, we did observe that lower mean Glu level post treatment with an NMDAR antagonist combined with lurasidone predicts better antidepressant response in BP-D, consistent with prior findings (21) in MDD when ketamine was employed. This relationship was seen despite a lack of significant between-treatment group differences for symptoms or ^1^H MRS outcomes, and was not seen after treatment with lurasidone alone or placebo. Of course, the small sample size precluded an adequately powered statistical analysis.

In a secondary finding, we demonstrate tolerability and potential efficacy of acute, high-dose DCS in BP-D when combined with lurasidone and no reports of psychotomimetic symptoms when receiving this medication combination. While the analysis was limited by the small sample, statistically significant improvement in depression was seen after an acute dose of DCS+lurasidone, but not with lurasidone alone or placebo. Similarly, the degree of clinical response was comparable to our previous open label findings of efficacy over 8 weeks of DCS combined with atypical antipsychotics (27).

We previously proposed that an increase in Glu may be a stress response because it is most robust in the placebo and healthy control groups (19, 21). While meta-analysis of medicated MDD patients indicate lower levels of Glx, when medication status is considered, the data indicate that medicated MDD has lower Glx or glutamate and untreated MDD may have elevated levels (43). Studies of BP-D have reported higher Glu levels (44–46), as have studies in other relatively treatment resistant populations such as postpartum depression (47). Similarly, a large mega-analysis found that while medial frontal cortex Glu and Glx are generally lower in schizophrenia compared to healthy controls, higher Glu and Glx levels were associated with more severe symptoms and lower levels were associated with antipsychotic treatment (48).

While our small sample size limited the ability to assess between group differences in the Glu response between scan days, we replicate our previous findings with ketamine (21), finding that DCS combined with lurasidone, appears to diminish the Glx or glutamate response and this effect correlates with degree of antidepressant effect. A reduced stress response is consistent with preclinical studies, indicating that NMDAR antagonist related antidepressant response may produce a resilience effect (49). Similarly, putative glutamatergic treatments in schizophrenia also appear to reduce NMDAR antagonist induced glutamate increases (50). Thus, we have previous proposed that elevated Glu or Glx may be a marker of depressive illness severity (51), and a reduction is an indicator of antidepressant response to NMDAR antagonists.

In our previous study of DCS alone in healthy controls (27), we found a positive peak at ~35 min post-dose (23 ± 5% increase). In the present report of BP-D patients, we found a small decrease in Glu after DCS+lurasidone treatment, consistent with a blunting of the elevation seen in other studies including those employing the NMDAR antagonist ketamine. Similar to our ketamine study (21), this blunting was correlated with degree of antidepressant effect.

The use of target engagement biomarkers early in drug development can facilitate dose selection and initial proof-of-mechanism assessments (50, 52–54). While the present report was not designed to assess dose response, our results do further support that target engagement at the NMDAR and the NMDAR glycine site, as measured by ^1^H MRS, is necessary for antidepressant response. Exemplary of this, a recent study of treatment resistant depression (55), found neither antidepressant nor ^1^H MRS Glu changes in response to AV-101, a competitive antagonist at the NMDAR glycine site. A subsequent study of AV-101 in healthy controls found evidence for a dose response for AV-101 using the auditory steady state response (56), and suggested that higher doses may be needed.

Our study has several limitations, and we emphasize its presentation as a pilot study. The small sample is the principal study limitation. A second concern is the potential carry-over effects from Scan 1 treatment with lurasidone or pre-study medications, especially concomitant mood stabilizers or those with a long half-life such as fluoxetine. Although the discontinuation of other antipsychotics and fluoxetine lowered the blood and brain levels of these medications, this was not for long enough to allow them to wash out of the brain completely. Consequently, although this step reduced the potential impact of such medications, it did not eliminate the possibility of an effect. These limitations are minimized by the lack of baseline Glu and MADRS differences between Scan 1 and 2, and only one subject was taking fluoxetine pre-study. Furthermore, potential variability in Glu from the use of two scanners or pre-study medication differences was minimized because we focused on the acute percentage change in Glu post study drug administration within each day for each subject, not absolute Glu values.

Finally, we focused on Glu in the present report instead of the composite measure of Glx by taking advantages of data acquisition parameter TE = 120 ms, which is optimized for Glu separation (28, 29) and spectral fitting prior knowledge obtained from simulated model spectra (30, 38). While this optimized our ^1^H MRS sequence for Glu measurements, the spectral overlapping between Glu and Gln might result in a “competition” or a “compensation” between them in the fitting, limiting the accuracy of Gln. Therefore, the variation of Glx may be smaller than that of Gln itself but may still larger than Glu. For this reason, we did not focus on Glx, nor report Gln. This limits the direct comparison to our Glx results in prior studies (21, 27). Better spectral fitting methods need to be developed to improve the fitting of Glu, Gln, and Glx.

In conclusion, our preliminary pilot results are consistent with our previous work. Attenuation of the Glu response being correlated with antidepressant response to NMDAR antagonists requires replication in a larger, multi-dose, controlled study. If replicated, this biomarker may prove to be a method for screening NMDAR antagonists for antidepressant potential.

## Data Availability Statement

The raw data supporting the conclusions of this article will be made available by the authors, without undue reservation.

## Ethics Statement

The studies involving human participants were reviewed and approved by NYSPI IRB. The patients/participants provided their written informed consent to participate in this study.

## Author Contributions

ZD, JM, and JK had full access to all of the data in the study and take responsibility for the integrity of the data and the accuracy of the data analysis. JK, ZD, MM, and JM: substantial contributions to conception and design. JK, ZD, MG, ML, VW, T-HC, TS, and JM: acquisition, analysis, or interpretation of data. JK, T-HC, JM, and ZD: drafting of the manuscript. JK, T-HC, JM, ML, and ZD: critical revision of the manuscript for important intellectual content. All authors reviewed the final submission and gave final approval of the submitted version.

## Conflict of Interest

JK reports having received consulting payments within the last 24 months from Alphasights, Charles River Associates, Medscape, Putnam, techspert.io, Third Bridge, MEDACorp, Parexel, GroupH, Simon Kucher, ECRI Institute, ExpertConnect, Parexel, Schlesinger Group, CelloHealth, Acsel Health, Strafluence, Guidepoint, L.E.K. and System Analytic. He serves on the MedinCell Psychiatry and Karuna Mechanism of Action (MOA) Advisory Boards. He has conducted clinical research supported by the NIMH, Sunovion, Roche, Alkermes, Cerevance, Corcept, Takeda, Taisho, Lundbeck, Boehringer Ingelheim, NeuroRX and Teva within the last 24 months. JK was a co-investigator on a study that receives lumeteperone and reimbursement for safety testing for an investigator-initiated research from Intra-Cellular Therapies Inc. He owns a small number of shares of common stock from GSK. JM receives royalties for commercial use of the C-SSRS from the Research Foundation for Mental Hygiene. The remaining authors declare that the research was conducted in the absence of any commercial or financial relationships that could be construed as a potential conflict of interest.
